# 50 Years of the Journal of the Brazilian College of Surgeons: History, Evolution and Future Prospects

**DOI:** 10.1590/0100-6991e-20243862EDIT01-en

**Published:** 2024-12-12

**Authors:** GERSON ALVES PEREIRA, ANDREIA CRISTINA FEITOSA DO CARMO, JULIA CASTRO NEVES, RAMIRO COLLEONI

**Affiliations:** 1 - Universidade de São Paulo, Curso de Medicina de Bauru - São Paulo - SP - Brasil; 2 - Universidade Federal de São Paulo, Biblioteca do Campus São Paulo - São Paulo - SP - Brasil; 3 - Colégio Brasileiro de Cirurgiões, Departamento de Publicações - Rio de Janeiro - RJ - Brasil; 4 - Universidade Federal de Sao Paulo, Escola Paulista de Medicina - São Paulo - SP - Brasil

**Keywords:** Open Access Publishing, Medical Societies, Scientific Dissemination Publications, Publicação de Acesso Aberto, Sociedades Médicas, Publicações de Divulgação Científica

## Abstract

This article celebrates the 50th anniversary of the continuous publication of the Journal of the Brazilian College of Surgeons (RCBC), revisiting its trajectory from the beginning to the present day. RCBC has evolved from a nationally relevant publication to a journal with international impact, constantly adapting to changes in editorial and scientific practices. This article presents an analysis of the major milestones, editorial changes, and innovations that have cemented RCBC as a prominent scientific vehicle. In addition, it discusses future strategies to maintain editorial quality and increase the visibility of the journal in the global scientific scenario.

## RESUMO

Este artigo celebra os 50 anos de publicação contínua da Revista do Colégio Brasileiro de Cirurgiões (RCBC), revisitando sua trajetória desde o início até a atualidade. A RCBC evoluiu de uma publicação nacionalmente relevante para uma revista com impacto internacional, adaptando-se constantemente às mudanças nas práticas editoriais e científicas. Este artigo apresenta uma análise dos principais marcos históricos, as mudanças editoriais, e as inovações que têm consolidado a RCBC como um veículo científico de destaque. Além disso, discute as estratégias futuras para manter a qualidade editorial e ampliar a visibilidade da revista no cenário científico global.

## INTRODUCTION

The Journal of the Brazilian College of Surgeons (RCBC) plays a key role in the dissemination of surgical knowledge in Brazil. Since its creation, it has followed the evolution of surgical and scientific practice, contributing to the training and updating of health professionals. This publication, which began with a national focus, gradually gained international projection, and is now indexed in several prestigious scientific databases. This article aims to celebrate RCBC’s 50-year history, highlighting the main achievements and challenges faced, as well as exploring the projections for the future of the journal.

### History of the journal - origins and early years

The Journal of the Brazilian College of Surgeons (RCBC) started in 1930 as the “Bulletin of the Brazilian College of Surgeons”, gradually becoming a traditional and relevant medical journal, although published with varying periodicity. The current name came in 1967 by decision of the Brazilian College of Surgeons (CBC) National Directory and has been published bimonthly and regularly since 1974. A single continuous annual volume online has been adopted since January 2020. During its first decades, the number of submissions was modest, reflecting a scientific landscape still in development in the country. The journal has undergone a series of adaptations, evolving from a national publication to a platform that serves the international scientific community.

### Consolidation and indexations

From the 2010s onwards, RCBC began to focus on editorial quality and scientific rigor, resulting in its indexation in renowned databases, such as SciELO, LILACS, SCOPUS, and PUBMED. This expansion was accompanied by an increase in the number of articles received, as well as by the growing participation of reviewers with different expertise, which contributed to the diversity and depth of topics covered. 

In the last three years, we have updated our editorial policies and practices to maintain international indexes and, recently, the full availability of articles published since 2009 was approved, in digital preservation of PubMed Central - PMC (https://www.ncbi.nlm.nih.gov/pmc/journals/4491/).

RCBC will start using another complementary indicator, which is “altmetrics”. This concept has gained strength, as it conveys that the measurement of impact by citations of research and its authors cannot be restricted to publications aimed at the scientific community. It also proposes to consider media and channels aimed at a wider audience, spaces considered non-traditional in scientific communication - or “alternative”, hence the name - bringing to the debate the repercussion of research on society. In other words, the popularization of social networks on the internet and the approximation of academia with traditional and digital media are included in the articles count.

### Recent changes and innovations

#### Review of editorial policies and processes

In recent years, RCBC has adopted new policies to ensure quality and transparency in publications, including the use of similarity tests and a greater emphasis on the methodological rigor of the articles received. In the submission of manuscripts by the authors, a checklist was created for analysis of the mandatory documentation of manuscripts submitted to RCBC: a) Authors’ form, signed individually by each author, b) Authors’ register compatible with the information contained in the Form and in the manuscript (the same authors who individually signed the form must be in the manuscript), c) Opinion of the Ethics Committee regarding the submitted article (when applicable), having the title similar to the article, d) Equator Checklist (when applicable), individual for each type of article, e) Text of the manuscript in Portuguese, when the author is Brazilian, f) Check if there are tables, graphs, and figures and if they were sent individually, g) If any item(s) are missing, return them to the authors to correct and correct the submission, and h) Only perform the similarity test after all documentation is adequate.

These changes resulted in a safer review process, disciplining authors and significantly reducing the time between submission and publication​.

### Analysis of production indicators over time

By monitoring the indicators of submission, review, and approval/failure of articles submitted to RCBC in the last decade, particularly in the last two years, it was possible to zero the number of delayed manuscripts awaiting review, reduce the average review time of approved manuscripts, even increasing the total number of reviews and reviewers per article. 

There is a clear improvement in the quality of the articles we have received, which has motivated our reviewers to play their roles within an adequate deadline. Logically, some areas are still deficient, and we have taken measures to improve them.

The approach to the initial evaluation of manuscripts under review has been very pedagogical, instructing authors in the necessary adjustments for the best quality of the method and the scientific text.

### Digital Platform and Technologies

In response to the demands of increasingly digital science, RCBC will launch a new website with innovative features, such as videos recorded by authors about their articles, allowing for greater interaction between readers and researchers. In addition, the migration to a new platform enabled a more intuitive and effective submission and review process, contributing to experience improvement, both for authors and reviewers.

### Search for investment sources

For the first time in its history, RCBC submitted a funding proposal for the CNPq/CAPES Call No. 30/2023 - Editorial Program. The purpose of this call was to support proposals aimed at encouraging the editing and publication of highly specialized Brazilian scientific journals in all areas of knowledge, to significantly contribute to the country’s scientific and technological development and innovation. The objectives of this call were:

a) to support proposals for editing and publication of highly specialized Brazilian scientific journals in all areas of knowledge;

b) to expand access to the results of scientific research conducted in the country; 

c) to contribute to the scientific and technological development of the country.

We met all eligibility criteria, having granted the amount of R$ 20,000.

### Engagement with the Scientific Community

The training course for reviewers was set up as one of the strategies adopted to increase the engagement of the scientific community. 

The target audience will be the editors and reviewers of the Journals of the Brazilian College of Surgeons, as well as other interested parties. 

The objective of the course will be to train scientific reviewers and editors to act efficiently and ethically in all stages of the submission and evaluation process of manuscripts of the RCBC, ensuring the quality and integrity of the publications.

The course was organized for the knowledge and preparation of reviews and editing of scientific articles, with various sequential discussion topics, with the presence of experienced and renowned tutors in their themes.

The course will be held using a flipped classroom with synchronous and asynchronous activities. A main text (didactic material prepared by the tutor), a recorded video with the presentation on the theme, and bibliographic references will be available as a complementary library. This way, each topic will have a recorded presentation for prior study provided the previous week and synchronous online discussion the following week with the invited tutors, according to the course agenda.

At each moment of the theme discussion schedule (biweekly meetings by videoconference), the topics of prior study are defined through didactic materials prepared by the guest tutors, as well as by recorded video classes. At the time of classes each week, there will be an online discussion with the tutors of the selected topics, according to the scheduled presentation themes.

The Table below shows the schedule of the course that will start in the first quarter of next year.

[Table t1]

**Table t1:** 

Period of prior study	Modules	Themes	Synchronous meeting date	Tutors
Day 1	Opening	Course objectives and expectations	Day 1	Pedro Portari (CBC), Ramiro Colleoni (UNIFESP), Andreia Carmo, and Gerson Alves Pereira Júnior (USP)
Day 2 a 15	Module 1: Introduction to the Editorial Process	1.1. Overview of scientific publication with emphasis on surgery 1.2. Structure and organization of the Journal of the Brazilian College of Surgeons 1.3. Roles and responsibilities of editors and reviewers	Day 15	Cristiano Xavier de Lima (UFMG) Gerson Alves Pereira Júnior (USP)
Day 16 a 30	Module 2: Ethics and Integrity in Scientific Publishing	2.1. Copyright and Licensing 2.2. Plagiarism, self-plagiarism, inappropriate citations, and use of generative AI 2.3. Retractions and corrections 2.4. Conflict of interest 2.5. Peer review: types, processes, and best practices (20 minutes)	Day 30	Andreia Carmo (UNIFESP) Gerson Alves Pereira Júnior (USP) Luciano - Editora Cubo
Period of prior study	Modules	Themes	Synchronous meeting date	Tutors
Day 31 a 45	Module 3: Manuscript Evaluation - part 1 Module 3: Manuscript Evaluation - part 2	3.1. Analysis of the different types of articles 3.2. Structure and formatting of articles 3.3. Analysis of methodology, results, and conclusions 3.4. Quality and relevance criteria	Day 40 Day 45	Marcus Kodama (USP/SP) Marcus Kodama (USP/SP)
Day 46 a 60	Module 3: Manuscript Evaluation - part 3	3.5. Good practices in reviewing articles and preparing opinions 3.6. Databases and research/bibliographic references 3.7. Analysis of integrative, scoping, and systematic reviews	Day 60	Maria de Lourdes Biondo-Simões (UFPR/PR) Pedro Lourenção (UNESP) Melissa Moraes (Pouso Alegre/MG)
Day 61 a 75	Module 4: Communication with Authors and Reviewers	4.1. Preparation of opinions and constructive feedback 4.2. Deadlines and time management 4.3. Dispute and Disagreement Resolution 4.4. Privacy and confidentiality	Day 75	Rafael Mozeto (Cubo) Andreia Carmo (UNIFESP)
Day 76 a 90	Module 5: Tools and Resources for Reviewers and Editors	5.1. Editorial management software and platforms 5.3. Plagiarism and formatting checker tools 5.4. Artificial Intelligence tools in submission and review	Day 90	Rafael Mozeto (Editora Cubo) Andreia Carmo (UNIFESP)
Day 95	Closure	New flowchart for the role of the editor-in-chief, associate editors, and reviewers	Day 95	Ramiro Colleoni (UNIFESP) and Gerson Alves Pereira Júnior (USP)

This initiative aims to form a more robust and qualified collaboration network, essential for advancing the quality of published articles.

### Current impact and future projections

#### Growing visibility and scientific impact

RCBC has consolidated its position as a reference in the national scientific scenario, with increasing quality indicators. The goal of indexing RCBC in the Web of Science (WOS) reflects the ambition to further raise the journal’s prestige, expanding its influence both in Brazil and abroad. The improvement in CiteScore over the past few years is indicative of RCBC’s growing impact..

### Future

#### Innovation and Sustainability

For the coming years, RCBC projects the implementation of sustainable publishing practices and the encouragement of open science. Commitment to constant innovation, such as the adoption of emerging technologies and the development of new open access policies, are essential to ensure the relevance of RCBC in an increasingly competitive and dynamic scientific landscape.

The next and decisive step for the Journal of the Brazilian College of Surgeons, aiming at its consolidation and commitment to excellence and relevance in the global context of scientific research and publication, guided by the principle of open science, is the strategic objective of integrating the journal into the renowned Web of Science database. The inclusion of RCBC in WOS is crucial, especially considering that few Latin American journals are indexed in the Journal Citation Reports (JCR), which highlights the underrepresentation of our research in the international scenario. With this, RCBC will immediately achieve an impact factor and entry into WOS will not only increase the visibility and credibility of our studies, but will also foster research and international collaboration, highlighting the quality and relevance of the science developed in our country. This step is crucial to ensure the continuity of our influence and leadership in the field of surgery. For inclusion in WOS, the selection process must be considered, which uses a uniform set of 28 criteria, divided into two main categories: 1) editorial quality and 2) impact. The Editorial Quality category comprises twenty-four criteria focused on best editorial practices and at the journal level. These criteria assess the integrity of the peer review process, the transparency of editorial policies, the regularity of publication, among other fundamental aspects that support trust in scientific publication. On the other hand, the Impact category includes four additional criteria dedicated to measuring the impact of the journal in its field, using citation activity as the primary indicator, aiding in the identification of journals that are most influential in their respective fields.

To achieve this goal, we must follow a roadmap of criteria defined by WOS, implementing concrete measures that demonstrate our commitment to editorial and scientific quality, which include:

### Innovation and Accessibility

A new website for RCBC has been ready, designed to be more than just a showcase of articles. We want it to be a platform for engagement and collaboration, where interactivity and accessibility allow the dissemination of knowledge in a broad and effective way. In addition, the authors of the articles will be invited to record a video with a systematized and short format to disseminate their approved article. This will not only increase our national and international visibility, but also reinforce the importance of our scientific contribution to global and local communities.

### Editorial Excellence

We have meticulously planned a significant improvement of our editorial process. The adoption of a new manuscript management system, in collaboration with Editora Cubo, will provide a new and updated platform for articles submission and evaluation. This system will allow a more effective and transparent management of submissions and reviews, aligning with the international standards required for qualification in WOS. The efficiency and integrity of this system are essential to raise the standards of quality and rigor our scientific community needs to evolve into.

### Ethical and Professional Guidelines

The new guidelines for authors reflect our commitment to ethics in research and publishing. In line with SciELO recommendations, these guidelines are a fundamental pillar to maintain confidence in the integrity of the works we publish, ensuring that each article has a valuable contribution.

### Diversity and Excellence in the Editorial Board

Expanding the editorial board to include international members is a strategic decision that goes beyond simple diversification. It is a conscious effort to enrich our editorial and scientific perspective, which is fundamental to maintaining RCBC’s relevance and quality. The inclusion of experts from various parts of the world brings with it a vast body of knowledge and a wide network of international collaborations, which are crucial to boost both the quality and impact of our publication.

We understand that changes in the national editorial board may raise concerns, especially when they involve the departure of old members. However, it is important to highlight that turnover on the editorial board, although not a common routine in Brazil, is a healthy and necessary practice that we must implement. It allows the journal to renew itself and adapt to changes and advances in the scientific field, in line with internationally used standards. In addition, the turnover facilitates the inclusion of new perspectives and specializations, reflecting emerging trends and the current needs of the global scientific community. Therefore, in each cycle, we seek to integrate new members who can contribute with updated views and knowledge. This not only reinforces our commitment to excellence and innovation, but also ensures that our journal continues to be a vehicle for the dissemination of high-level science, respecting and promoting integrity and ethics in scientific publishing. This strategy of renewing and diversifying the editorial board is essential for RCBC to maintain its leadership position and continue to expand its impact in the world of surgery. The achievement of an impact factor in WOS will be another historic milestone for RCBC, raising not only our visibility, but also reaffirming our commitment to innovative research. This inclusion is a step towards ensuring that our articles reach a global audience, amplifying our work’s impact and usefulness.

### New journal

The idea is to keep RCBC rising in national and international indexes and this greatly raises the threshold of the articles that must be approved. However, we need to conclude a series of articles that have been submitted and need many adjustments and longer revisions, especially methodological issues, to be able to be approved for publication. Thus, in discussion on the subject since the Congress of the Brazilian Association of Scientific Editors at the end of 2023 in Foz do Iguaçu/PR, we realized that the best alternative would be to create another journal to drain the national publication and decide on its name, mission, and scope. We also noted that, among other national journals, there is a vacuum of journals focused on teaching surgery and, more broadly, medical education and other areas of health, as well as urgencies and emergencies, both traumatic and in surgical clinics. The idea is to have a reformulated proposal for the Journal of Case Reports, with a broader, more current, and defined format of the Case Series Report (Clinics, with a focus on diagnosis and therapy, anatomopathology, and imaging), with the possibility of thematic symposia, privileging the multidisciplinary focus and stimulating integration with new teaching, information, and communication technologies. The currently most endorsed name is Revista de Educação em Cirurgia, Emergência e Saúde - Surgical, Emergency and Health.

## CONCLUSION

As it celebrates five decades of continuous publication, the Journal of the Brazilian College of Surgeons reaffirms its commitment to scientific excellence and the knowledge dissemination. The history of RCBC is marked by a constant adaptation to changes in the scientific world, remaining current and relevant to the national and international surgical community. With an eye to the future, the journal seeks to consolidate its position as a quality reference in scientific publishing, ready to face the challenges and take advantage of the opportunities of a constantly evolving scenario.

We must adopt individual and institutional measures that will ensure the highest quality in national scientific publications, including the training of human resources (undergraduate, medical residency, and postgraduate), training of advisors, public and private policies to encourage scientific projects, maintenance of active and functional research groups in the different areas of surgery in all regions of the country, and training of reviewers.

None of this would have been possible without the valuable participation of our reviewers from the most varied areas of surgery, who have made earnest efforts to meet the deadlines necessary for RCBC to further improve its indicators of speed of manuscript evaluation.
